# Digital Trespass: Ethical and Terms-of-Use Violations by Researchers Accessing Data From an Online Patient Community

**DOI:** 10.2196/11985

**Published:** 2019-02-21

**Authors:** Emil Chiauzzi, Paul Wicks

**Affiliations:** 1 PatientsLikeMe, Inc Cambridge, MA United States

**Keywords:** ethical issues, social media, data sharing, privacy, informed consent, data protection, data anonymization

## Abstract

With the expansion and popularity of research on websites such as Facebook and Twitter, there has been increasing concern about investigator conduct and social media ethics. The availability of large data sets has attracted researchers who are not traditionally associated with health data and its associated ethical considerations, such as computer and data scientists. Reliance on oversight by ethics review boards is inadequate and, due to the public availability of social media data, there is often confusion between public and private spaces. In addition, social media participants and researchers may pay little attention to traditional terms of use. In this paper, we review four cases involving ethical and terms-of-use violations by researchers seeking to conduct social media studies in an online patient research network. These violations involved unauthorized scraping of social media data, entry of false information, misrepresentation of researcher identities of participants on forums, lack of ethical approval and informed consent, use of member quotations, and presentation of findings at conferences and in journals without verifying accurate potential biases and limitations of the data. The correction of these ethical lapses often involves much effort in detecting and responding to violators, addressing these lapses with members of an online community, and correcting inaccuracies in the literature (including retraction of publications and conference presentations). Despite these corrective actions, we do not regard these episodes solely as violations. Instead, they represent broader ethical issues that may arise from potential sources of confusion, misinformation, inadequacies in applying traditional informed consent procedures to social media research, and differences in ethics training and scientific methodology across research disciplines. Social media research stakeholders need to assure participants that their studies will not compromise anonymity or lead to harmful outcomes while preserving the societal value of their health-related studies. Based on our experience and published recommendations by social media researchers, we offer potential directions for future prevention-oriented measures that can be applied by data producers, computer/data scientists, institutional review boards, research ethics committees, and publishers.

## Introduction

According to the Pew Research Center [1], the majority of Americans use social media websites such as Facebook (68%) and YouTube (75%), with roughly a quarter to one-third using other sites such as Snapchat, Instagram, LinkedIn, and Twitter. The sheer volume of data arising has proved to be an inviting target for both social good and ethically questionable practices alike. Social media data have driven important public health research, including monitoring disease outbreaks [2], predicting health risk behaviors [3], accessing hard-to-reach populations [4], health promotion [5], user health-communication patterns [6], and mutual medical data sharing between patients [7]. Some researchers have adopted a more participatory approach by engaging high-risk groups such as drug users to detect trends and encourage harm reduction [8]. The analysis of these data has ushered in a variety of innovative analytic techniques such as natural language processing, network analysis, deep learning, and geolocation to provide further insight into these large datasets [9].

With such a rapidly evolving landscape, this area has been no stranger to ethical controversy [10,11]. Ethical questions have arisen in highly publicized cases such as the Facebook social contagion study [12,13], the release of an OKCupid dataset of 70,000 users [14], and most recently, the use of 50 million user profiles on Facebook by Cambridge Analytica during the 2016 US presidential campaign [15]. In each of these cases, large quantities of user profile data compromised user privacy or manipulated users through targeted messaging.

Academic reviews suggest that there is “widespread neglect” of ethical considerations by social media researchers [16], such as inadequate informed consent, lack of researcher boundaries, reposting of personally identifiable content, and deliberate misrepresentation or deception [16] [17,18,19]. A recent study found that online searches of verbatim Twitter quotes found in journal articles can be tracked back to individual users 84% of the time [17], despite users’ lack of awareness of this sharing, resistance to being studied, and desire to consent to these practices [18,19]. Some researchers misrepresent themselves or engage in deception to engage with social media participants [20]. Many researchers assume that social media data are in the public domain, obviating the need for consent altogether [21].

There may be several reasons for these challenges. First, researchers conducting studies in the United States may believe that approval by institutional review boards (IRBs) is sufficient for addressing ethical considerations. A recent review of 156 academic studies mining social media found that ethical considerations were limited only to minimum requirements for IRB approval, rather than broader ethical considerations (eg, privacy, public and private spaces, and original contexts for providing data) [16]. Only 13 of 156 (8%) studies mentioned ethical considerations beyond IRB approval. Researchers relying on the US Department of Health and Human Services (USDHHS) Common Rule guideline to bypass informed consent when research does not involve an intervention or uses “existing data sets” inadequately address these ethical concerns [22], which may be further amplified in stigmatized conditions such as mental illness [23]. Second, IRB members may lack consensus among themselves on the IRB review process in social computing research, the need for informed consent, their own regulatory obligations, and criteria for evaluating social media projects on a case-by-case basis [24]. Third, considering social media users as just another class of traditional “human subjects” misses the mark. Critics also suggest that traditional definitions of terms such as “human subject,” “informed consent,” privacy, ownership of data, terms of use, and private and public settings are too narrow for online contexts [21]. Finally, current regulations such as the USDHHS Common Rule emphasize risk mitigation at the initial stages of research—study design and data collection—rather than at later stages that involve access to and dissemination of data [25]. Informed consent collected at a single point in time may not account for the “drift” that occurs in a participant’s willingness to share data [26].

Outside of the United States, there are a wide variety of national research ethics governing bodies and over 1000 laws, regulations, and standards that provide oversight for human subjects research in 130 countries [27]. The rigor of ethical review varies widely across countries. In Europe, ethics review is generally stringent and managed through national bioethics agencies, health ministries, food and drug safety organizations, national research committees, etc [27]. Ethical review processes in countries such as China are less well developed, with a lack of standardization in operating procedures, professional ethics training, protection of vulnerable groups, and privacy safeguards [28]. In both of these scenarios, issues of privacy, data trustworthiness, and consent have yet to be resolved, even with the advent of the European Union General Data Protection Regulation (GDPR) [29]. Research ethics committees (RECs) often lack the expertise to evaluate technical standards, methodologies, data ownership, and group-level ethical harms in big data studies [30]. Taken together, these issues suggest that international ethical review frameworks continue to be highly challenged by the current dynamic social media research environment.

Second, accessing and de-identifying social media data is not difficult. Data transgressions can be enabled by the ready availability of user data combined with the dissemination of “scraping” technologies that allow easy extraction [31]. Data scraping and de-anonymizing can be accomplished by individuals with no more than basic programming and statistics skills [32]. Unfortunately, privacy has been considered a “binary value”—either public or private [33]—rather than a continuum [34]. While some researchers assume that information shared in public spaces is inherently available for public consumption and may be used without consent, it is important to respect the nature of the data, collection context, and user expectations [33]. Identifiability should not be regarded as a binary value (either “public” or “private”), but as a continuum based on the nature and extent of the data [33]. Attempts at de-identification are a necessary but insufficient to ensure safe use of data [34], with some researchers warning that true de-identification is a “false promise” [35]. Re-identification has been accomplished with relatively limited data available such as Netflix subscriber movie ratings [32] or simple demographics [36].

Third, the perception that big data are somehow “objective” and can be analyzed independent of context is an illusion [37,38,39]. Social media users post information for reasons differing widely from what researchers may imagine. For example, within the PatientsLikeMe platform [40], patients adopt a broader definition of “treatments” than clinicians and researchers. For patients, treatments may include “pets” and “handicapped parking stickers” just as much as medications, medical procedures, and therapies. Faulty data assumptions and researcher biases may cascade into poorly built algorithms that lead to ultimate inaccurate (and possible harmful) conclusions, termed by O’Neil [41] as “weapons of math destruction.” It is important not to dissociate the data from the *people* behind them [33]. Even when aggregate data are used and no individual identification has been made, researchers need to be sensitive to the potential psychological and behavioral consequences of findings (particularly with stigmatized or vulnerable groups) as well as the scale and generalizability of conclusions [23,42]. There is a risk of type I error when findings are overgeneralized [43], thus requiring more mixed methods and longitudinal data gathering [42].

Fourth, health research has traditionally been conducted by researchers trained in human subject ethics and overseen by established ethics panels. However, the recent growth of “big data” sets in health has attracted computer science researchers who may be less well versed or monitored with regard to key ethical issues [10]. Wright [44] warns that many computer scientists are skirting the ethical traditions of medical and social science professionals, who abide by guidelines such as the Belmont Report [45] and the USDHHS Common Rule [46]. Buchanan et al [47] suggest that computer science researchers “may not fully understand or believe that their projects align with the same ethical concerns that pertain to human subjects, such as the minimization of risk or harm to individuals, confidentiality, privacy, or just recruitment methods.”

Several questions arise in this context. How do these ethical violations occur? How are these violations discovered and remedied by data producers? Most importantly, what corrective actions can and should be taken to prevent violations that compromise the privacy of social media users? In order to address these questions, we share four cases involving ethical and terms-of-use violations that highlight the four challenges described above. These violations involved the use, interpretation/misinterpretation, and dissemination of patient self-reported data and forum posts available at PatientsLikeMe [40]. In this manuscript, our goal is to utilize these cases as a springboard to protect patient privacy while finding ways of meeting investigators’ legitimate public health research objectives.

## Case Studies: Real-World Experiences From an Online Health Community

The following four cases provide examples of ethical and methodological issues that arise when researchers gather social media data without observing the website’s terms of use. These cases have been selected as representatives of the breadth of issues encountered over 12 years (typically, at a rate of one or two per year) at PatientsLikeMe, an online patient community devoted to research (Textbox 1).

Each of the cases illustrates a different set of ethical problems. We have applied the health-related research ethics guidelines created by the Council for International Organizations of Medical Sciences (CIOMS) in conjunction with the World Health Organization [48] as a primary framework for these cases.

For the purposes of this paper, we will distinguish the ethical violations from terms-of-use violations, which represent a lack of adherence to website-specific policies or approval to conduct research-related activities. The lack of attention to terms of use by prospective users accessing various websites and apps has been well documented [49] and should be distinguished from the ethical violations noted earlier. Terms-of-use violations may include participation in ways that do not conform to the purpose of the forum, posting false content, unauthorized scraping of data, or a lack of authorization to conduct research by the data producer. There is certainly potential for these concepts to overlap, particularly on websites that involve the sharing of personal health information. Table 1 describes the types of violations as well as CIOMS guidelines that apply to the cases in this manuscript.

Because we aimed to remain transparent, we emailed the prepublication manuscript to the researchers represented in Cases 2-4 below (Case 1 has already been publicized in the national press). After providing 1 month for responses but receiving none, we moved forward with the final manuscript. We have not named specific researchers or papers in Cases 2-4 in order to preserve their anonymity.

### Case 1: Large-Scale Data Scraping by Commercial Market Researchers

#### Background

In a well-publicized 2009 incident reported in the Wall Street Journal [50], staff at the company Nielsen Media sought to understand how patients with mental health conditions talked about the company. The company created an unauthorized account on PatientsLikeMe and used automated “scraper” software to begin copying open-text discussion data from the message board forums. In total, they harvested about 5% of the mood disorder forum’s qualitative discussion content for an undisclosed commercial client. Our team detected the scraping software, suspended the account (and three others linked to it) shortly after it was initiated, and emailed the company to ask them to stop.

#### Relevant Terms of Use and Ethical Guidelines

Because this was considered “market research,” no IRB was involved. For market researchers, the level of ethical oversight is not the same as that for academic researchers in most studies. However, professional bodies such as the Market Research Association state that members should “Protect the rights of respondents, including the right to refuse to participate in part or all of the research process,” among other guidelines [54]. Market researchers may need to develop their own standards related to health-data gathered online or endorse existing guidelines. For example, the Association of Internet Researchers recommends that researchers obtain consent from either participants individually or community owners [21]. Harvesting sensitive data from people with mental health issues also warrants consideration of vulnerable populations; without proper procedures in place to ensure data were handled correctly, there is a risk of re-identification. Scraping only the visible data (as opposed to accessing a full dataset) risks drawing spurious or biased conclusions.

#### Response

We emailed the company with a cease-and-desist letter. PatientsLikeMe sent a private message to its entire membership describing the incident and wrote a blog post about it. As a result, about 200 members decided to close their accounts. Six months later, reporters at the Wall Street Journal investigated the story as part of a series looking at scraping activity on the Web, and the incident was reported on the newspaper’s front page [50].

**Description of PatientsLikeMe.**

PatientsLikeMe is an online community of over 600,000 people living with about 2900 medical conditions including amyotrophic lateral sclerosis, mood disorders, HIV, and rare diseases [51]. As part of the membership, individuals who are interested in joining the site are asked to review our user agreement [52] and privacy policy [53]. The user agreement describes acceptable lawful use, inappropriate posting practices, and restriction of content use within the site. The privacy policy provides clear and transparent communication about data as well as rights to see, correct, and delete data; get notified if data are stolen; and request that data processing stop.

The PatientsLikeMe privacy policy is written in plain language and allows patients to review, correct, or delete their data. Patients may self-report their conditions, treatments, symptoms, and patient-reported outcome measures (reporting as much or as little as they like) and are able to look at aggregated reports to help decide how they might better manage their condition. Most individual “profiles” are only viewable to other members, who must “log in” to the site after registering with an email address, while some aggregate data reports are viewable from the “logged out” part of the website. Although patients are comfortable anonymously sharing their data with vetted researchers [29], there are many ways in which an uninformed external researcher could misinterpret the way data are collected or be unaware of known biases that are familiar to our internal researchers. In addition, anyone entering “fake data” can potentially trigger negative consequences for data quality and, potentially, even patient safety.

PatientsLikeMe has adopted this model because patients lack access to information that can affect their treatment decisions. Sharing “real world” data allows patients, providers, and researchers to collaborate in evaluating current treatment effectiveness, gaps in treatment, and potential new and better treatments. This collaboration can speed the pace of research and improve health care delivery. To facilitate this mission, PatientsLikeMe is funded through investment, as well as commercial and academic research partnerships, rather than advertising or member fees. Because of the serious nature of health data, PatientsLikeMe has been committed to applying these data responsibly toward patient-centered goals and implementing a “data for good” philosophy. Responsible big data research seeks soundness and accuracy of data while maximizing good and minimizing harm [33].

**Table 1:** Case violations and Council for International Organizations of Medical Sciences guidelines.

Violation type		Case 1 - Commercial scraping	Case 2 - De-anonymization of forum user	Case 3 - Fake profile data	Case 4 - Multiple scraper bots	Relevant CIOMS^a^ guideline number
**PLM^b^ terms-of-use violations**						
	Not a patient, caregiver, health care professional, or visitor with legitimate reasons to participate^c^	✓	✓	✓	✓	7, 22
	Posting false content^d^			✓		4, 11
	Use of any robot, spider, scraper, or other automated means to access the site or content^e^	✓	✓		✓	7, 12, 22
	Lack of research authorization by PLM^f^	✓	✓	✓	✓	7, 8, 9, 10, 22, 25
**Ethical violations**						
	De-identifying patient data in any way		✓			4, 11, 14, 15, 22
	Inadequate/no informed consent	✓	✓		✓	9, 10, 12, 22
	False identification or misrepresentation			✓	✓	4, 22
	Verbatim use of user posts		✓			4, 11, 12, 14, 15, 22

^a^CIOMS: Council for International Organizations of Medical Sciences.

^b^PLM: PatientsLikeMe.

^c^PLM user agreement: “To become a member and access the area on this Site reserved for members (the ‘Member Area’), PatientsLikeMe requires that you are either a (a) diagnosed patient of the particular community you are joining or a parent or legal guardian acting for such a patient who is under 18 years of age or incapacitated, (b) caregiver for a patient eligible to join such community, (c) health care professional (e.g. doctor, nurse, health researcher, etc.), (d) guest with legitimate, non-commercial reasons to participate in the community and who agrees to respect the privacy and preserve the dignity of all community participants or (e) guest as authorized by a PatientsLikeMe member or employee.”

^d^PLM user agreement: “Members shall not post or upload any information or other content on the Site that (a) is false, inaccurate or misleading; (b) is obscene or indecent; (c) infringes any copyright, patent, trademark, trade secret or other proprietary rights or rights of publicity or privacy of any party; or (d) is defamatory, libelous, threatening, abusive, hateful, or contains pornography.”

^e^PLM user agreement: “You may not use any robot, spider, scraper, or other automated means to access the Site or content or services provided on the Site for any purposes.”

^f^PLM user agreement: “Please note that under our terms of service, you are not permitted to capture or utilize data from within the site nor to solicit members through our forums or private message to take part in your study.”

#### Resolution

In the Wall Street Journal article, a company representative stated, **“**It was a bad legacy practice that we don't do anymore...It’s something that we decided is not acceptable, and we stopped.” Corrective efforts included upgrading our automated scraper-detection software, clarifying how commercial researchers could contact PatientsLikeMe for authorization, determining which actions are permissible and not permissible on the site, and sustaining communication with our members about the implications for their data and further participation on the site.

### Case 2: De-anonymization of Individual Forum Members

#### Background

Around 2014, computer science researchers at a European university developed an algorithm that could be used to de-identify highly sensitive medical data, which individuals might choose to share on social networks in order to reduce their risk of personal identification. The system involved automated methods for determining the “identifying information content” of a given piece of data (ie, “I’m a woman living with a mental health condition for the past two years” vs “my name is Susan and I was diagnosed with bipolar disorder in Boston on June 2, 2016”). In order to illustrate their approach, they provided in their manuscript a verbatim text quote from a member discussing how they came to be diagnosed with HIV. The authors published their study, whereupon a Google Scholar Alert notified us that the research had taken place.

#### Relevant Terms of Use and Ethical Guidelines

No formal ethics review was conducted, which may have contributed to the oversight. In terms of accessibility, while this story was “shared online,” it was on a private profile accessible only to other patients logged into the site. Searching for the verbatim text within the logged-in area of PatientsLikeMe quickly identified the member concerned. Although de-identification is never foolproof (and indeed, this was the point of the study itself), if the patient had decided to change his/her mind and delete the data or close their PatientsLikeMe account, the quote and the patient’s association with it could have persisted permanently within the scientific literature. CIOMS guideline 22 on the use of data obtained from the online environment states, “When researchers use the online environment and digital tools to obtain data for health-related research they should use privacy-protective measures to protect individuals from the possibility that their personal information is directly revealed or otherwise inferred when datasets are published, shared, combined or linked [55].” Additional considerations should have been given, as HIV is a highly stigmatized condition.

#### Response

During other similar incidents in the past, reaching out solely to the authors or their institutions often failed to yield a response. As a result, we emailed the authors and the journal editor with our concerns to ensure this issue would be dealt with appropriately.

#### Resolution

As no specific patient data were mentioned in the papers, no data were scraped from the site. The focus was a theoretical algorithm, and all parties quickly realized their error. A partial retraction was agreed upon to replace the verbatim quote with a synthetic quote. PatientsLikeMe notified the member concerned. Although CIOMS guideline 22 speaks to research in the online environment, the guidance is general instead of recommendations for best practices for every platform. More specific advice for preventing risk to patients can be found from NatCen’s Social Research guidance, which recommends “(Test) the traceability of a tweet or post and (take) responsible steps to inform the user and protect their identity, if desired. Best practices include paraphrasing instead of verbatim quotes and not using an individual’s handle/user name.”

### Case 3: Researcher Misrepresentation and Fake Profile Data

#### Background

Researchers at a European university secured a grant to investigate the extent to which users of social networks thoroughly read and consider the “terms of use” of social networks like PatientsLikeMe. To test this in controlled conditions, 20 students were asked to register accounts on PatientsLikeMe and complete fake data from a prespecified set of instructions. Focus groups held with the students later revealed that most of them had not read the terms of use. The authors published their study, whereupon a Google Scholar Alert notified us that the research had taken place 10 months before. Both grant funding and REC approval were sought and granted for this study, despite the lack of a “letter of support” from PatientsLikeMe as a potential collaborator.

#### Relevant Terms of Use and Ethical Guidelines

Deceptive practices such as researchers misleading participants about their identity are never acceptable, and we were surprised that an REC had approved such activity. In our case, researchers prompted students to enter fake data into a system requiring log-in, which is used by patients, regulators, and health care professionals to guide practice and conduct medical research. CIOMS guideline 1 states, “Although scientific and social value are the fundamental justification for undertaking research, researchers, sponsors, research ethics committees and health authorities have a moral obligation to ensure that all research is carried out in ways that uphold human rights, and respect, protect, and are fair to study participants and the communities in which the research is conducted. Scientific and social value cannot legitimate subjecting study participants or host communities to mistreatment, or injustice.”

#### Response

We emailed the authors, REC, and funders with our concerns. The researchers stated that they did not think they needed permission for a “publicly available forum” and emphasized that the focus of their research was not medical but informational, focusing on the “terms of use” rather than the data of the PatientsLikeMe members themselves. A number of discussions and arguments had to be put forward to explain to the researchers *why* this behavior was wrong in the first place; one analogy we used was that while students *could* pretend to be sick patients in a hospital waiting room in order to conduct research on the clarity of signage within the institution, this would quickly be understood as unethical.

The researchers thought their activities were “outside the logged-in” parts of the site (which they were not) and that students had never re-accessed their accounts after the initial study (which they had). The REC agreed that entering false data was suboptimal behavior, admitted to confusion around some of the complex technical issues surrounding online research, and agreed this was an area they would learn more about in future. The funding body claimed that as the institution had its own REC, they had no further responsibility to check that the permissions were in place.

#### Resolution

As no specific patient data were mentioned in the papers, no data were scraped from the site, and the focus was indeed the understanding of the “terms of use.” We agreed with the authors that a partial retraction in rescinding the name of PatientsLikeMe from their papers would be enough, along with assurances that this would not happen again. We also agreed that our terms and conditions could benefit from clarification. Between this experience and the recent enactment of the European GDPR, work is underway currently to clarify patients’ rights in terms of privacy and access to their data and to make explicitly clear that just because patients share their data within the community, it does not grant researchers the right to use the data. When making a determination of whether a community is public or private, the researcher should consider the availability of information to the general public, member perceptions of privacy, sensitivity of content, record permanence, and the intended audience of the study [20].

### Case 4: Repeated Scraping Through Multiple Accounts

#### Background

Computer science researchers at an Asian university sought to build a neural network capable of determining whether side effects that members were attributing to a treatment they were taking might, in fact, be symptoms of their condition; for example, “trouble sleeping” might be caused by their *depression* rather than *a drug* they were taking. In order to gather test data, they created an account on PatientsLikeMe and began “scraping” data from patient profiles with automated software. When our security systems were tripped by the software activity, their account was closed. Over the following 2 weeks, multiple, seemingly related, accounts were created, many from “disposable” email accounts, in order to continue scraping, which were closed as soon as we identified them. With data from over 5000 users, they prepared a manuscript for a computer science conference to be presented a year later, comparing the reported experience of patients to a third-party data source and describing their algorithm. The authors published a preconference proceeding, whereupon a Google Scholar Alert notified us that the research had taken place 10 months before.

#### Relevant Terms of Use and Ethical Guidelines

Multiple CIOMS guidelines appeared to be breached, including respect for rights (guideline 1, no permission or consent was requested), balancing individual risks and benefits to participants (guideline 4, no steps were taken to minimize harm to patients), community engagement (guideline 7, the data were gathered covertly), consent (guidelines 9 and 10, no consent was requested or exempted), use of health data (guideline 12, patients’ response to treatment was scraped and analyzed), vulnerable persons (guideline 15, the focus included members with severe mental health issues), online environment (guideline 22, the researchers did not inform the community), and ethics committee review (guideline 23, this work did not undergo formal ethics review). The researchers did not appreciate that using a logged-in account was crossing a boundary nor that active shut down of their accounts by our security team was a “no entry” signal. In our discussion, the researchers appeared to feel that because the emphasis of their research was neural networks, they were “far” from medical data. More traditional medical researchers would have had to undergo quite considerable ethical oversight, consent, and data privacy policies to access similar data from a hospital or insurer. Building systems that used such algorithms to judge the soundness of a patient report risked diminishing the fidelity of patients’ lived experiences; many, if not most, patient experiences with disease and treatments cannot be found in medical texts, and few medical researchers would assume that divergence meant that the patients were automatically “wrong.”

#### Response

We emailed the authors, conference chairs, and chair of their department with our concerns and requested full retraction of the paper, identification of all scraper accounts, and deletion of all data. The researchers stated that they had only accessed “public” parts of the site, denied having used multiple scraper accounts, said that the data had been held securely, and requested they be allowed to anonymize the data source. In mitigation, they claimed that the paper had received positive peer reviews from the community. Initially, the conference chairs were against retraction based on their judgement that no “material harm” had been done to PatientsLikeMe, that scraping the data was technically easy for a researcher to perform, and that it was unclear whether any laws had been violated. However, further careful investigation by our security team revealed that over 50 “bot” accounts were created from the same rather narrow geographical region during a time period consistent with the conduct of the methods detailed in the paper. On further discussion, the authors admitted that “maybe” an intern had done this. However, scientific record keeping was lacking, as no systematic records had been kept to verify this.

#### Resolution

The authors apologized and deleted all locally held data. The conference chairs accordingly decided that the authors had not been truthful, and therefore, the study was retracted from the conference proceedings. PatientsLikeMe notified the members concerned. Because the authors were not forthcoming about their activities, our security team had to exhaust significant resources in determining which accounts were bots and which users’ data had been accessed, and in refuting the authors’ claim. In addition, significant management resources were consumed communicating with the authors and other parties, and communication resources were used in messaging the affected users.

## Discussion

### Overview

These case studies highlight the broad challenges that arise when researchers gather social media data without prior authorization. The current literature on social media ethics emphasizes issues such as “terms of use,” “informed consent,” and “data privacy,” but the practical implications of these infractions creates ripple effects on patients and the staff responsible for protecting their data. Researchers may gather data to satisfy their scientific goals but should balance these with potential adverse effects on patients, the company affected by the terms of use violation, and the validity of their research enterprise. The lack of informed consent and respect for privacy deprives potential participants of choice regarding the use of their data. Once personal health data have been accessed in an unauthorized or unethical manner, the wide availability of powerful search tools create additional threats to patient privacy.

We believe there are many ways in which the analysis of social media data can contribute to the public good as well as inform individuals about ways to improve and maintain their health. However, the lack of equitable data access, underlying biases in data interpretation, and inadequate transparency between those who provide and those who analyze data risks squanders the many potential advantages of algorithmic decision making [56]. Throughout these cases, we believe that researchers based their treatment of study participants’ data on several false assumptions that violated a number of ethical guidelines.

#### Faulty Assumption 1: “The Internet” Is Not Subject to Ethical Review

Throughout our experiences, we perceived the sense that data (and the “social media users” contributing them) are less worthy of respect or protection when users participate online as opposed to when the same “patients” receive care in a brick-and-mortar health institution like a hospital. To add to the matter, members of ethics review boards may not consider social media studies to be human subject research under current legal definitions and may not believe that data scraping requires informed consent [57]. In our view, social media and “big data” research is not ethically exceptional and should be treated in the same manner as traditional forms of research [57]. Of the cases reported here, only Case 2 obtained ethical approval, and even then, the behaviors exhibited fell short of what we could consider ethical. Terminology may cloud matters, as existing guidelines may confine themselves only to “biomedical” or “medical” fields, which may lead some researchers to exclude their projects from ethical oversight on the basis that their focus or their branch of study is computer science, business, or design. However, CIOMS [48] uses the broader term “health-related research” to encourage greater inclusiveness rather than focusing on researchers’ occupation or training. Online contexts should be compared to offline analogues to highlight potential considerations that may affect informed consent; if it was not acceptable to do something in a hospital waiting room, doing it on the internet does not absolve researchers of responsibility. We believe that interpreting the USDHHS Common Rule for “existing data set” as “free access to any health data set on the Internet” is a faulty assumption.

#### Faulty Assumption 2: Social Media Spaces Are “Public”

In our discussions with individuals involved in the cases reported here, we encountered a lack of cultural sensitivity to the “perceived privacy” of individuals choosing to share information within a “closed network” as opposed to an open forum. It is probably best to take a conservative approach and consider that any content requiring an email for access may not be considered public by a site’s users.

Where trespasses were acknowledged, they were claimed to be justified by good intentions. For example, while few would argue in favor of the potentially good intentions of gathering and analyzing social media posts in Case 1 to try and understand mental health problems, such good intentions do not act as blanket absolution from ethical considerations such as consent, privacy, de-identification, or minimization of harms. In the real world, reading and analyzing the diaries or written correspondence of patients with mental health problems would not be deemed acceptable even if they were left unsecured.

#### Faulty Assumption 3: Data Can Be Analyzed Independent of Context

Although large datasets may appear alluring by their sheer scale, in practice, they can introduce larger errors of interpretation by inspiring false confidence in the conclusions drawn. In Case 4, the researchers were unaware that there was a host of additional contextual data recorded about how patients had multiple comorbidities and understood the purpose of their medications or that they may have been using some treatments for off-label purposes rather than their standard indications [59]. The absence on their team of trained health professionals also obscured important context about the relationship between a condition’s symptoms and the common side effects of treatments used for the condition. Without understanding the sampling of a data set, the limits of meaningful questions and interpretations may not be observed [37]. Scientifically, data scraping without context may result in potentially inaccurate algorithms that may get reported and reused in application, leading to potentially harmful consequences [41]. Our discussions with researchers revealed a general lack of care and rigor that would be of scientific concern even without the ethical considerations. We explained the importance of understanding the context and structure of the data that were scraped in order to produce meaningful scientific results and requested a retraction of questionable findings and interpretations to avoid contaminating the literature.

#### Faulty Assumption 4: Computer Science Research Does Not Need to Abide by Health Research Guidelines When It Is Only Accessing “Data”

While computer science researchers were responsible for only Case 4 reported here, computer science practitioners are responsible for the bulk of our other unreported cases, confirming Wright’s [38] assertions that the field needs to adjust its practices before further incidents undermine their social license to practice. Computer scientists are “largely focused on the care and feeding of electronic devices” and may have different conceptions of what constitutes a “human subject”—a living person or data that are representative of a living person [42]. Involving computer scientists on ethics review boards may be an effective way of encouraging ownership of ethics issues from the inside out as well as assuring more technology expertise in medical and other studies. This would also encourage more complete paper trails when untangling ethics transgressions.

### Appropriately Resolving Terms-of-Use Violations

We have shared our experiences, in part, to guide other practitioners in the field. Unfortunately, the effects on data reporting may be difficult to detect and may not be caught until publications and conference papers appear. The resolution of the scientific inaccuracies and communications, as well as deletion of scraped data, often required difficult conversations over extended periods. We recommend that data producers develop their own standard operating procedures and hold practice scenarios when responding to violations.

For instance, because substantial time and effort are devoted to research planning, execution, and publication, a recently published or in-process journal article represents a considerable “sunk cost.” As a result, researchers, funders, conference organizers, and journal editors may apply pressure to data producers to “allow” publications to proceed with corrections rather than retract findings. Over the course of the cases experienced by our team, nearly every supervisor, institution, conference chair, or publisher challenged in the case of a violation first asked (politely) for clemency, forgiveness, “retrospective consent,” or even “post-hoc ethical approval.” Rather than adopt a punitive philosophy, we respectfully reminded these researchers of our responsibilities to patients who are our members and from whom we have earned social license to use and maintain their data responsibly. However, having policies and prepared communications in place early on would reduce the burden on staff members who may find such interactions challenging.

### Limitations

Our report contains several limitations. First, the authors are employees of a for-profit company and therefore have a conflict of interest in “protecting” network data. We hope to encourage similar experiences by others in the academic or nonprofit sphere to share their experiences. Second, the cases reported here are relatively brief and due to our desire to preserve anonymity where possible, there is little additional detail for interested readers. Third, as a complex and emerging area, our conclusions are necessarily editorial rather than evidence based. For example, future work could survey social network users whose data have been shared without their consent. Finally, the individuals described herein may not feel they have an adequate “right to reply”; we would welcome divergent views on the topics we have outlined here.

### Future Directions: Prevention Rather Than Cure

Based on the need to maximize benefits while limiting potential harm in social media research, we believe that there are several potential strategies that can be pursued. First, rationales such as social media data use for “public benefit” and “public interest” need to be carefully defined [34]. Investigator transparency is as critical in social media research as in traditional forms of research; researchers should disclose their presence, not misrepresent themselves, and be truthful about the risks and benefits of their studies [48,58]. Researchers should exercise extreme caution in adapting or combining data sets for potentially invasive purposes. Common sense strategies such as avoiding the reuse of verbatim quotes should be adopted. It is therefore critical for researchers to understand the nature of the data source that they are accessing. As part of ethics protocol submission, investigators should certify that they are complying with the terms of use of the targeted research websites or justify to ethics review boards why their methodologies fail to comply [57]. In addition, ethics review boards should include members with strong knowledge of online research and computer science methodologies, so that applications for ethical approval can be vetted more carefully [47].

When data have been collected without authorization, there should be standard operating procedures developed and followed with regard to how data obtained without authorization should be managed, deleted, and verified. University information technology departments could take a lead in this regard. Further attention to ethical issues in computer and data science training and conduct may help prevent the violations discussed in this paper while recognizing the value of important research questions. Data producers (such as PatientsLikeMe) and data scientists can enhance each other’s work if an appropriate dialogue can take place. Data producers can adopt a proactive stance by finding ways to curate and expand access to views of their data (such as through application programming interfaces), so that important scientific research can be encouraged while minimizing ethical and terms-of-use violations. In order to meet the needs of computer science and other researchers, PatientsLikeMe has started investigating ways to provide tools for researchers to interrogate data sets in order to yield insights with less risk to member privacy.

Such strategies would only be the beginning of addressing social media privacy challenges, but we welcome further enhancement of and feedback on these ideas. A group of data scientists recently reported on a crowdsourced “Hippocratic Oath for Data Science” [60] that calls upon their peers to “Ensure that all data practitioners take responsibility for exercising ethical imagination in their work, including considering the implication of what came before and what may come after, and actively working to increase benefit and prevent harm to others.”

## Abbreviations

CIOMS: Council for International Organizations of Medical Sciences
GDPR: General Data Protection Regulation
IRB: institutional review board
REC: research ethics committee
USDHHS: United States Department of Health and Human Services
